# Bystander Effects in Osteoblasts and Osteoclasts: Comparison Between X- and Proton-Irradiation

**DOI:** 10.1016/j.ijpt.2025.101203

**Published:** 2025-09-16

**Authors:** Noriyuki Okonogi, Yukako S. Otani, Keisuke Otani, Paola Divieti Pajevic, Yunhe Xie, Nicolas Depauw, Harald Paganetti, Jan Schuemann, David T. Miyamoto, Kathryn D. Held, Aimee L. McNamara

**Affiliations:** 1 Department of Radiation Oncology, Massachusetts General Hospital/Harvard Medical School, Boston, MA 02114, USA; 2 QST Hospital, National Institutes for Quantum Science and Technology, Chiba 263-8555, Japan; 3 Department of Radiation Oncology, Juntendo University Graduate School of Medicine, Tokyo 113-8421, Japan; 4 Department of Translational Dental Medicine, Goldman School of Dental Medicine, Boston University, Boston, MA 02118, USA

**Keywords:** Radiation-induced bystander responses, Bone cells, Irradiation, X-ray, Proton beam irradiation

## Abstract

**Purpose:**

This study investigated radiation-induced bystander responses (RIBR) in osteoblasts and osteoclasts and compared x-rays with protons regarding RIBR effects.

**Materials and Methods:**

MC3T3-E1 subclone 4 (preosteoblasts), differentiated MC3T3-E1 (osteoblasts), and differentiated RAW264.7 (osteoclasts) were irradiated with x-rays (225 kVp) and protons with a linear energy transfer of −2.3 keV/µm. A trans-well insert coculture system, allowing the transfer of soluble factors without contact between irradiated and nonirradiated cells, was used to study medium-mediated RIBR. The range of intended dose was 0.5 to 2 Gy for both x-ray and proton-irradiation. The RIBRs of nonirradiated cells were evaluated based on the expression of 53BP1 at 1 h postirradiation and micronuclei assay at 72 h or later.

**Results:**

RIBR was evident in bystander cells regardless of the irradiated/nonirradiated cell combination. The percentage of 53BP1-positive cells was 3.2% to 4.2% in controls and 8.6% to 11.7% in bystander cells (*P* < .05). Similarly, binucleated cells with micronuclei were 3.7% to 6.0% in controls versus 13.1% to 15.4% in bystanders (*P* < .05). No differences in RIBR were observed based on radiation type or dose.

**Conclusion:**

This study provides preliminary evidence that RIBR occurs in osteoblasts and osteoclasts, irrespective of cell combination, dose, or radiation type, suggesting that both x-rays and protons may induce comparable bystander effects in bone-related cells.

## Introduction

Radiation therapy (RT) plays a crucial role in managing various types of cancer, including breast cancer (BC). Along with surgery and systemic therapies, RT is vital for the treatment of BC.^1^ Postoperative RT consistently improves local control, with greater efficacy in BC patients at higher risk of local recurrence.^1^ Therefore, RT for BC patients is highly recommended unless the patient is pregnant or has a specific genetic disorder.^2^, ^3^ With appropriate treatment, BC generally has a favorable prognosis, making effective management and prevention of adverse events (AEs) essential.^4^, ^5^, ^6^, ^7^, ^8^, ^9^ Among these AEs, studies have shown that radiation dose to cardiac structures increases the risk of major cardiac events.^10^, ^11^

Proton beam therapy, due to its dose localization properties compared to x-rays, reduces cardiac radiation exposure.^12^ However, a recent phase II study of proton beam therapy for BC patients indicated a higher incidence of rib fractures compared to conventional x-ray RT, despite its ability to minimize cardiac radiation exposure.^13^ While rib fractures are not fatal, they significantly impact the patient’s quality of life and should be prevented. With the increasing use of proton beam therapy for BC patients,^12^ a thorough understanding of the effects of irradiation, including proton beam therapy, on bone has become a critical clinical issue.

The skeleton is a metabolically active organ that undergoes continuous remodeling throughout life. Bone remodeling involves the removal of mineralized bone by osteoclasts, followed by the formation of new bone matrix by osteoblasts, which subsequently becomes mineralized.^14^ Preclinical and clinical studies have shown that radiation causes bone destruction in a dose-dependent manner.^15^, ^16^ Therefore, it is essential to consider the effects of RT on both osteoblasts and osteoclasts to fully understand the comprehensive impact of radiation.

There have been reports of the direct effects of radiation on the bone cells.^17^, ^18^ However, when discussing the effects of ionizing radiation, attention should also be given to radiation-induced bystander responses (RIBR). RIBR refers to a phenomenon where nonirradiated cells exhibit radiation-like effects, as demonstrated in various cell types, tissue models, and in vivo studies.^19^, ^20^, ^21^ This phenomenon is mediated by mechanisms such as the diffusion of soluble factors and gap junction intercellular communication.^22^, ^23^, ^24^ However, it remains unclear whether RIBR occurs in bone cells or whether differences exist in RIBR between proton and photon beams in these cells.

Therefore, this study investigated whether RIBR occurs in osteoblasts and osteoclasts and whether there are differences in RIBR effects between x-rays and protons.

## Materials and methods

### Cell culture

MC3T3-E1 subclone 4 (ATCC), an osteoblast precursor cell line derived from mouse calvaria (the sex unspecified), and RAW264.7 (ATCC), a macrophage male murine cell line, were used in this study. Cells were cultured in Minimum Essential Medium (Thermo Fisher Scientific) supplemented with 10% fetal bovine serum, 100 U/mL penicillin, 100 μg/mL streptomycin, and 0.25 μg/mL amphotericin B, and grown at 37 °C with 5% CO_2_.

### Cell differentiation

**Figure 1:**
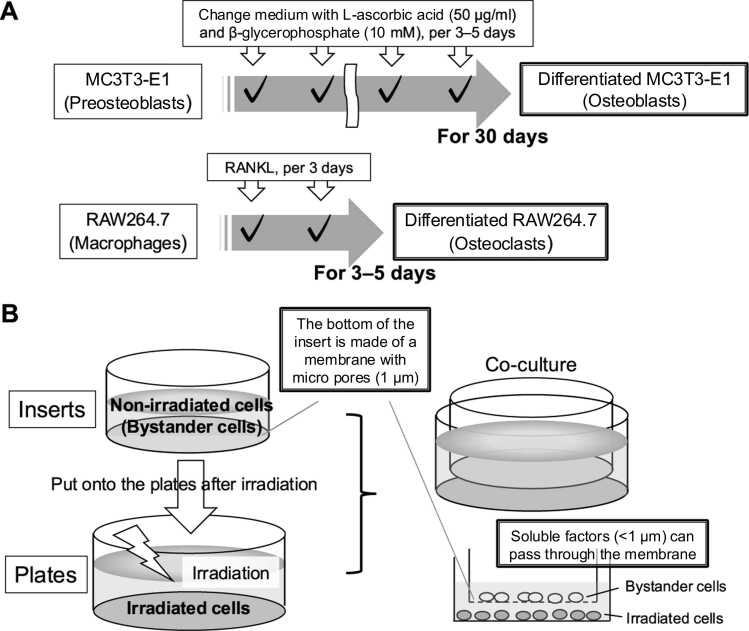
Experimental materials. (A) Showing how differentiation is induced in MC3T3-E1 and RAW264.7 cells. (B) The method of verifying the bystander effect and the structure of the items used for the experiment. **Abbreviation:** RANKL, receptor activator of nuclear factor kappa-B ligand.

MC3T3-E1 cells served as preosteoblasts, while their differentiated form was used as osteoblasts. Subconfluent MC3T3-E1 cells were cultured with 50 μg/mL L-ascorbic acid (Merck) and 10 mM β-glycerophosphate (Merck), refreshed every 3 to 5 days. Differentiation was confirmed using qPCR (Supplementary Data 1). RAW264.7 cells were differentiated into osteoclasts by adding 50 ng/mL receptor activator of nuclear factor kappa-B ligand (Shenandoah Biotechnology) every 3 days (Figure 1A).

### Irradiation

X-ray irradiation was conducted using an X-RAD 225 L irradiator without filtration (225 kVp, 1.93 Gy/min, Precision X-Ray). Proton-irradiation was performed at the Burr Proton Center using a clinical proton beam delivered at the spread-out Bragg peak. The LET at the target position was approximately 2.3 keV/μm, as estimated by Monte Carlo simulations using the TOPAS toolkit, which has been extensively validated for the relevant beamline.^25^, ^26^ The dose was delivered at a rate of approximately 0.5 Gy/s. In accordance with standard clinical practice, the proton dose was expressed in Gy (relative biological effectiveness), calculated by multiplying the physical dose by a relative biological effectiveness factor of 1.1.

### Coculture system for verification of radiation-induced bystander effect

A trans-well insert coculture system was used to investigate the effects of medium-mediated bystander signaling.^19^ As shown in Figure 1B, the bottom of the Falcon insert dish (Corning Inc) has a membrane with 1.0 µm pores at a density of 1.6 × 10^6^/cm^2^ to allow the passage of molecules. Thus, soluble factors that can cause bystander effects and are <1 µm in size can pass through the membrane. One day before experiments, round cover glasses with a diameter of 18 mm (Thermo Fisher Scientific) were put in an insert and well of a 6-well dish, then 1 × 10^5^ cells were seeded on the coverslips. After irradiation of cells in wells on the plates (Figure 1B), irradiated cells were cocultured with nonirradiated cells until the desired time for assessments. The culture medium in the irradiated wells was not changed before initiating coculture. This was done to preserve soluble factors secreted by the irradiated cells immediately following exposure. Therefore, the bystander cells were exposed to both active secretions from irradiated, metabolically intact cells and any radiation-modified components present in the medium. This approach is consistent with prior study,^19^ and it has been reported that medium irradiation alone does not induce bystander effects in the absence of living cells. Combinations tested included MC3T3-E1 with differentiated or nondifferentiated MC3T3-E1, and differentiated RAW264.7 with MC3T3-E1.

### Staining for p53 binding protein-1

Cells were fixed in 4% paraformaldehyde, permeabilized in 0.5% Triton X-100 solution, and blocked with 5% goat serum. Anti-53BP1 antibody (1:1000; Abcam) was applied overnight at 4 °C, followed by Alexa Fluor 488 secondary antibody (1:1000; Abcam). A minimum of 300 nuclei in at least 20 fields of view were counted for each sample in each experiment. Nuclei with at least 10 foci were considered positive cells/nuclei for 53BP1.

### Micronucleus assay

Cytochalasin B (Wako) was added postirradiation to the cultures to a final concentration of 1.5 µg/mL. Cells were incubated for 72 to 96 h, fixed with methanol: acetic acid (3:1, v/v). Cells were stained with 5 µg/mL 4′,6-diamidino-2-phenylindole solution (Thermo Fisher Scientific) for 10 min at room temperature. A minimum of 300 binucleate cells in at least 20 fields of view were counted for each sample in each experiment.

### Statistical analysis

Data, presented as means ± standard deviation from 3 independent experiments, were analyzed using unpaired t-tests or ANOVA for multiple comparisons. Levene’s test assessed variance equality. Analyses were performed with SPSS 27.0 for Mac (IBM), and *P* < .05 was considered significant.

## Results

Supplementary Data 1B shows the changes in mRNA induced by differentiation of MC3T3-E1 cells. When normalized by *18S ribosomal RNA* expression, *alkaline phosphatase* expression significantly decreased, while osteocalcin expression increased upon differentiation of MC3T3-E1 cells.

**Figure 2:**
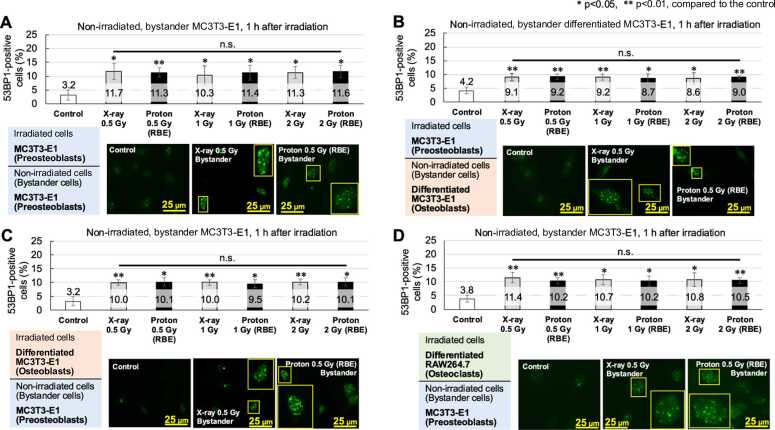
The results of the 53BP1 assay for nonirradiated, bystander cells. The 53BP1 assay results in nonirradiated (bystander) cells cultured with irradiated cells. The combination of irradiated and nonirradiated cocultured cells (=bystander cells) is shown in the upper left corner of each figure. The vertical axis represents the percentage of 53BP1-positive cells. Each sample was fixed 1 h after irradiation then stained. The results are expressed as the mean ± standard deviation of at least 3 independent experiments. White bars indicate controls (nonirradiated), gray bars indicate the results of x-ray irradiation, and black bars indicate the results of proton-irradiation. Nuclei with at least 10 foci were considered positive cells/nuclei for 53BP1. Yellow rectangles indicate 53BP1-positive nuclei. The larger rectangles are doubled-size images. *P < .05, **P < .01; compared to each control. (A) Irradiated cells, MC3T3-E1; non-irradiated cells, MC3T3-E1. (B) Irradiated cells, MC3T3-E1; non-irradiated cells, differentiated MC3T3-E1. (C) Irradiated cells, differentiated MC3T3-E1; non-irradiated cells, MC3T3-E1. (D) Irradiated cells, differentiated RAW264.7; non-irradiated cells, MC3T3-E1. **Abbreviations:** n.s., not significant; RBE, relative biological effectiveness.

**Figure 3:**
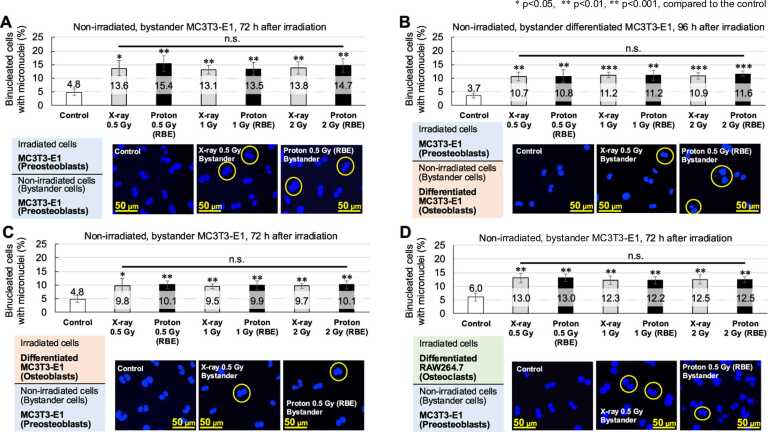
The results of the micronuclei assay for nonirradiated, bystander cells. The micronuclei assay results in nonirradiated cells cultured with irradiated cells. The combination of irradiated and nonirradiated cocultured cells (=bystander cells) is shown in the upper left corner of each figure. The vertical axis represents the percentage of binucleated cells with micronuclei. The difference in timing of evaluation is because of the differing proliferation rate of the cell lines. The results are expressed as the mean ± standard deviation of at least 3 independent experiments. White bars indicate controls (nonirradiated), gray bars indicate the results of x-ray irradiation, and black bars indicate the results of proton-irradiation. Yellow circles indicate binucleated cells with micronuclei. *P < .05, **P < .01; ***P < .001, compared to each control. (A) Irradiated cells, MC3T3-E1; non-irradiated cells, MC3T3-E1. (B) Irradiated cells, MC3T3-E1; non-irradiated cells, differentiated MC3T3-E1. (C) Irradiated cells, differentiated MC3T3-E1; non-irradiated cells, MC3T3-E1. (D) Irradiated cells, differentiated RAW264.7; non-irradiated cells, MC3T3-E1. **Abbreviations:** n.s., not significant; RBE, relative biological effectiveness.

Next, we examined the presence or absence of RIBR in various combinations of cell types. For differentiated RAW264.7 cells, which are multinucleated, the percentage of 53BP1-positive nuclei out of all nuclei was calculated. The extent of the bystander effect in nonirradiated cells, measured as the percentage of 53BP1-positive nuclei 1 h after irradiation, is shown in Figure 2. The percentage of 53BP1-positive cells for all cell types ranged from 3.2% to 4.2% in controls to 8.6% to 11.7% in bystander cells (*P* < .01 or <.05, compared to the control). No differences in bystander effects were observed based on radiation type or dose. RIBR at 72 h or later postirradiation was evaluated using the micronucleus assay (Figure 3). The percentage of binucleated cells with micronuclei ranged from 3.7% to 6.0% in controls to 13.1% to 15.4% in bystander cells (*P* < .001, <.01, or <.05, compared to the control) for all cell types. Similar to the 53BP1 results, no differences in bystander effects were observed based on radiation type or dose.

To evaluate the direct effects of irradiation on the donor cells, we assessed DNA damage and chromosomal instability in irradiated MC3T3-E1, differentiated MC3T3-E1, and RAW264.7 cells. At 1 h postirradiation, a dose-dependent increase in 53BP1-positive nuclei was observed in all cell types. Similarly, the frequency of micronucleated binucleated cells increased significantly at 72 or 96 h following irradiation. These results confirmed that irradiated cells remained viable and actively responded to radiation-induced damage, thereby supporting their potential role as active sources of bystander signals (Supplementary Figure S2.).

To further assess the functional consequences of radiation exposure, we conducted colony formation assays using MC3T3-E1 and differentiated MC3T3-E1 cells. As shown in Supplementary Figure S3, both cell types exhibited dose-dependent reductions in clonogenic survival following x-ray or proton-irradiation, with estimated D10 values indicating greater cell killing by proton-irradiation compared to x-rays, consistent with the known differences in LET between the 2 radiation types.

## Discussion

To our knowledge, this study represents the first exploratory report comparing the effects of bystander signaling in osteoblasts and osteoclasts using x-rays and protons. Our study revealed that RIBR occurs in several types of bone cells, regardless of the cell combination. The percentage of RIBR varied slightly depending on the cell line combination but was approximately 10%. So far, RIBR has been observed in many cell types, including lymphocytes, fibroblasts, endothelial cells, and tumor cells.^19^, ^27^, ^28^, ^29^, ^30^ However, this is the first report of RIBR in bone-related cells such as osteoblasts. While prior studies have demonstrated that radiation directly impairs bone cell viability and function,^17^, ^18^ our findings extend this knowledge by demonstrating that nonirradiated bone cells can also exhibit DNA damage and genomic instability via indirect bystander signaling. Although DNA damage does not always cause cell death, RIBR is probably not negligible when discussing the effects of irradiation on bone tissue. RIBR was observed irrespective of dose, cell combination, or radiation type. While our study did not evaluate RIBR in bone cells at doses below 0.5 Gy, previous studies, such as Yang et al,^31^ reported RIBR in fibroblasts at doses as low as a few cGy of proton beams. These findings suggest that the effects of lower-dose RIBR on bone warrant further consideration.

The present study found a comparable degree of DNA damage in bystander cells in the 53BP1 assay at 1 h postirradiation and in the micronucleus assay at 72 to 96 h postirradiation. Our direct assays of irradiated cells using 53BP1 and micronucleus assays further support this interpretation. Rather than undergoing immediate cell death, irradiated MC3T3-E1 and RAW264.7 cells demonstrated active DNA damage response and chromosomal instability, indicating that they remain metabolically active and capable of participating in intercellular signaling. This reinforces the plausibility that RIBR in our system reflects active biological communication rather than passive leakage. We did not analyze what triggered RIBR or the mechanism of RIBR in the present study. However, the experimental methods in our study point to RIBR by soluble factors that occur within 1 h after irradiation. Overall, the underlying mechanisms involved in RIBR signaling to unirradiated cells, as well as the cell signaling pathways in those cells, have been studied.^21^, ^22^, ^23^ However, there is still a lack of data on the mechanisms of bystander signal release from irradiated cells. Yokota et al^32^ showed a correlation between nitric oxide (NO) and RIBR using human fibroblast WI-38 cells. They demonstrated a high correlation between RIBR and NO concentration in the culture medium at 24 h postirradiation. Still, this study did not include information within 1 h postirradiation or 72 h postirradiation. It is still a conjecture, but perhaps multiple RIBR signals and cellular DNA repair mechanisms are dynamically and intricately involved. The mechanism of RIBR in bone cells is beyond the scope of this study, and further research on this topic is required.

Notably, our findings showing similar RIBR profiles between x-rays and protons appear inconsistent with clinical observations that proton therapy is associated with a higher incidence of rib fractures compared to photon therapy. This discrepancy may be due to differences in fractionation, dose distribution, and biological context. Clinical proton beams produce highly heterogeneous dose gradients, particularly at the distal edge of the Bragg peak, which may disproportionately affect rib structures adjacent to thoracic tumors. Moreover, our in vitro model does not replicate the mechanical loading, vascularity, or bone remodeling dynamics present in vivo. These limitations underscore the need for future research incorporating 3-dimensional bone models, animal studies, and clinical dose reconstruction analyses to bridge the gap between in vitro bystander data and clinical bone toxicity.

The present study includes several limitations. First, this study does not include results on osteocytes. Osteocytes are the cells residing within the bone matrix and comprising 90% to 95% of all bone cells.^33^ However, since the osteocytes were hypersensitive to Cytochalasin B, we could not perform the micronuclei assay in our experiments. Additionally, while RAW264.7 cells were differentiated into multinucleated osteoclast-like cells using receptor activator of nuclear factor kappa-B ligand, we did not evaluate the expression of osteoclast-specific molecular markers (eg, TRAP, Cathepsin K), which limits the confirmation of their functional identity. Because osteoclasts are terminally differentiated and nonproliferative, colony formation assays were not feasible, limiting our ability to assess long-term functional consequences of RIBR in these cells. A second limitation is the lack of data on the type of cell death after DNA damage has been observed. Confirmation of whether there is a difference in the morphology of cell death between x-rays and protons, especially in directly irradiated cells, may provide further clues to the mechanism of rib fracture in proton-irradiation. Third, our study does not explain the fact that the incidence of rib fractures increased with proton radiation compared to x-ray RT. Further investigation is needed to address these limitations. Fourth, our study did not evaluate RIBR at doses below 0.5 Gy. While the tested dose range (0.5-2 Gy) was selected to reflect clinically relevant exposures, previous studies have reported detectable bystander effects at much lower doses, including as low as a few cGy.^19^, ^31^ Due to resource limitations, we were unable to include these lower doses in the current study. Future studies assessing RIBR in the low-dose range will be necessary to fully characterize the dose-response relationship in bone cells. Fifth, we did not evaluate long-term outcomes beyond 96 h. While our assays captured early and intermediate responses to radiation exposure, further studies will be needed to determine the persistence, resolution, or biological consequences of RIBR at later time points.

In conclusion, this study provides preliminary evidence that RIBR can occur in osteoblasts and osteoclasts under in vitro conditions, with similar responses observed for both x-rays and protons. Future studies incorporating in vivo models and mechanistic analyses will be necessary to fully understand the implications of these findings in a clinical context.

## Ethics

This study did not use patient samples and did not require internal review board approval. All experiments were conducted in compliance with applicable laws and regulations.

## Funding

This work was funded by the Massachusetts General Hospital Radiation Oncology Loeffler Team Science Award. This work was also supported by Grants-in-Aid for Scientific Research, KAKENHI, Grant Numbers 23H02869, 23K27560.

## Author Contributions

**Noriyuki Okonogi:** Conceptualization, Methodology, Formal analysis, Investigation, Writing - Original draft, Visualization, Funding acquisition. **Yukako S. Otani:** Investigation. **Keisuke Otani:** Investigation. **Paola Divieti Pajevic:** Investigation, Data curation, Supervision. **Yunhe Xie:** Investigation. **Nicolas Depauw:** Resources, Supervision. **Harald Paganetti:** Resources, Supervision. **Jan Schuemann:** Investigation, Supervision. **David T. Miyamoto:** Supervision, Writing - Review and Editing. **Kathryn D Held:** Conceptualization, Methodology, Supervision, Project administration, Writing - Review and Editing. **Aimee L McNamara:** Conceptualization, Methodology, Supervision, Project administration, Funding acquisition. All authors commented on manuscript and have read and approved the final version.

## Declaration of Conflicts of Interest

The authors declare the following financial interests/personal relationships which may be considered as potential competing interests: Noriyuki Okonogi reports financial support was provided by the Ministry of Education, Culture, Sports, Science and Technology of Japan. Aimee L. McNamara reports financial support was provided by the Massachusetts General Hospital Radiation Oncology Loeffler Team Science Award. If there are other authors, they declare that they have no known competing financial interests or personal relationships that could have appeared to influence the work reported in this paper.

## Data Availability

The research data are stored in an institutional repository and will be shared upon request to the corresponding author.

## Appendix A Supplementary material

Supplementary Data 1. Quantitative real-time polymerase chain reaction (qPCR)

.

Supplementary Data 2. Direct effects of irradiation on bone cells. (A) Percentage of 53BP1-positive nuclei at 1 h postirradiation in MC3T3-E1, differentiated MC3T3-E1, and RAW264.7 cells. (B) Percentage of micronucleated binucleate cells at 72 or 96 h postirradiation. RAW264.7 cells do not divide, so micronucleus assays have not been performed. **P* < .05, ***P* < .01, ****P* < .001 compared to nonirradiated controls

.

Supplementary Data 3. Clonogenic survival of preosteoblasts and osteoblasts after irradiation. (A) MC3T3-E1 (preosteoblasts) and (B) differentiated MC3T3-E1 (osteoblasts) were exposed to x-ray or proton-irradiation at doses ranging from 0.5 to 6 Gy. Clonogenic survival was assessed by colony formation assay. Both cell types showed a dose-dependent decrease in surviving fraction following irradiation. The calculated D₁₀ values were 5.41 Gy (x-ray) and 4.35 Gy (proton) for preosteoblasts, and 5.73 Gy (x-ray) and 4.82 Gy (proton) for osteoblasts. The relative biological effectiveness (RBE) of protons compared to x-rays was estimated to be 1.24 for preosteoblasts and 1.19 for osteoblasts

## Footnotes

Appendix A Supplementary data associated with this article can be found in the online version at doi:10.1016/j.ijpt.2025.101203.
