# Supplementary material for: Bystander Effects in Osteoblasts and Osteoclasts: Comparison Between X- and Proton-Irradiation
Source: Int J Part Ther. 2025 Sep 16;18:101203. doi: 10.1016/j.ijpt.2025.101203 (PMC12508861; doi:10.1016/j.ijpt.2025.101203)
Supplement: Supplementary file 1 — Supplementary Data 1. Quantitative real-time polymerase chain reaction (qPCR) [file mmc1.pdf]

Supplementary Data 1.

A

Gene	Forward	Reverse
Alkaline phosphatase	5'-CCAACTCTTTTGTGCCAGAGA-3'	5'-GGCTACATTGGTGTTGAGCTTTT-3'
Osteocalcin	5'-CCGGGAGCAGTGTGAGCTTA-3'	5'-TAGATGCGTTTGTAGGCGGTC-3'
18S	5'-TCAAGAACGAAAGTCGGAGG-3'	5'-GGACATCTAAGGGCATCAC-3'

B

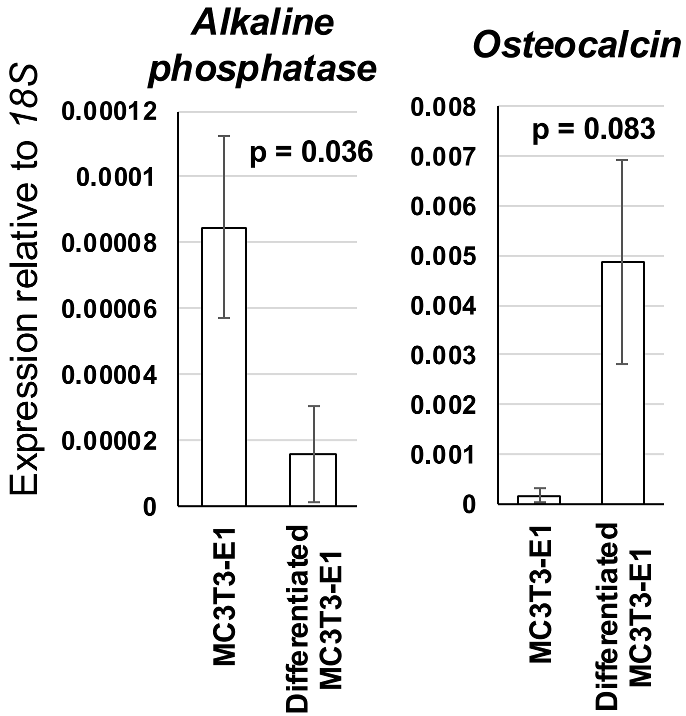

Quantitative real-time polymerase chain reaction (qPCR)

Total RNA was isolated from MC3T3-E1 cells using RNeasy Plus Kit (Qiagen, Hilden, Germany) according to the manufacturer's instructions. RNA (0.5–1 µg) was reverse transcribed into complementary DNA (cDNA) using PrimeScript RT reagent kit (Clontech, Takara Bio, Kusatsu, Japan) with a genomic DNA elimination step or qScript cDNA SuperMix (QuantaBio, Beverly, MA). Real time qPCR assay was subsequently performed with StepOne Plus (Applied Biosystems) using SYBR Green (Power SYBR, Thermo Fisher Scientific). The PCR conditions were as follows: one cycle of 95℃ (10 min), 50 cycles of 95℃, 58℃, and 72℃ (15 sec, 15 sec, and 30 sec, respectively), and one cycle of 95℃ (30 s), followed by a melting curve step (58℃ to 95℃ with an increment of +0.5℃, 1 min for each temperature). Data analysis was based on the comparative cycle threshold (ΔCT) method and expression of each gene was normalized to *18S ribosomal RNA expression*.
A shows the primer sequences for qPCR in this study. **B** shows the changes in mRNA induced by differentiation of MC3T3-E1 cells.
