# Supplementary figures and images for: Bystander Effects in Osteoblasts and Osteoclasts: Comparison Between X- and Proton-Irradiation
Source: Int J Part Ther. 2025 Sep 16;18:101203. doi: 10.1016/j.ijpt.2025.101203 (PMC12508861; doi:10.1016/j.ijpt.2025.101203)

Supplementary Data 2.

**A**

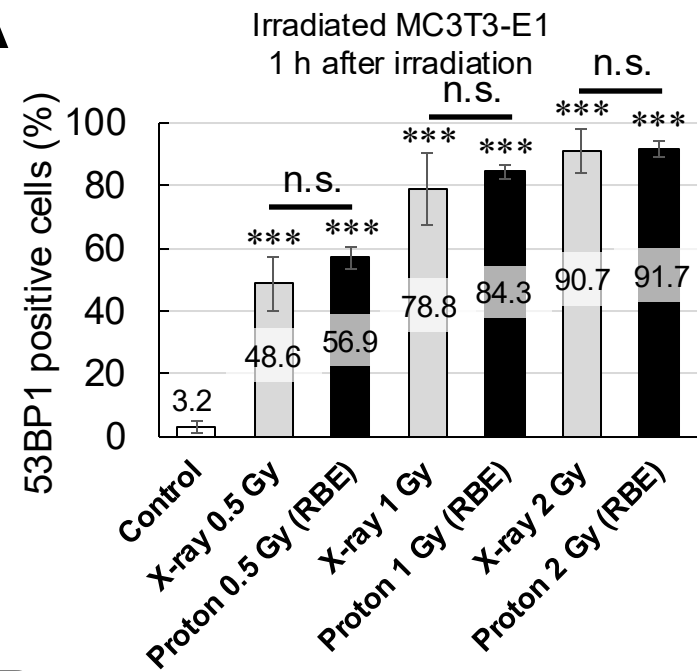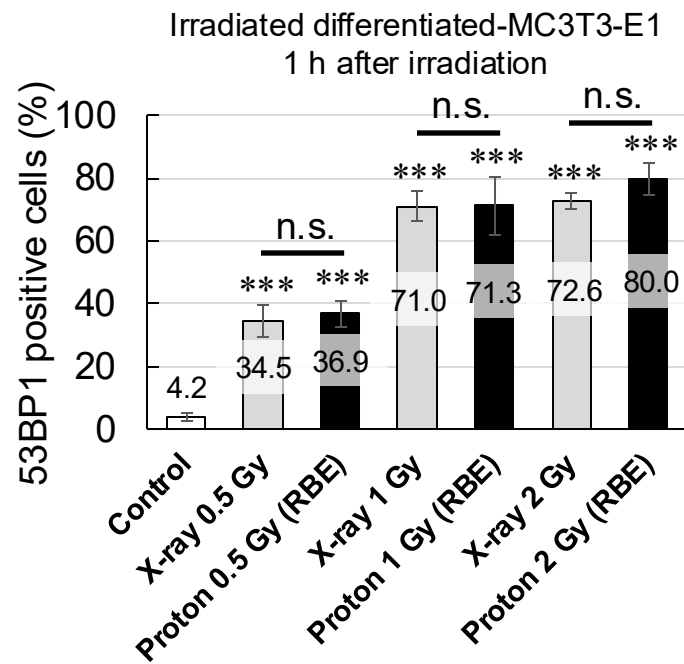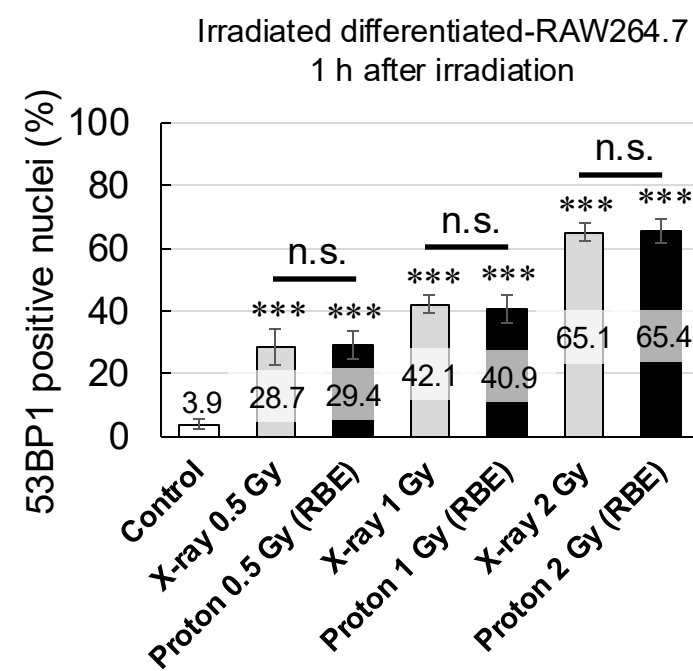

**B**

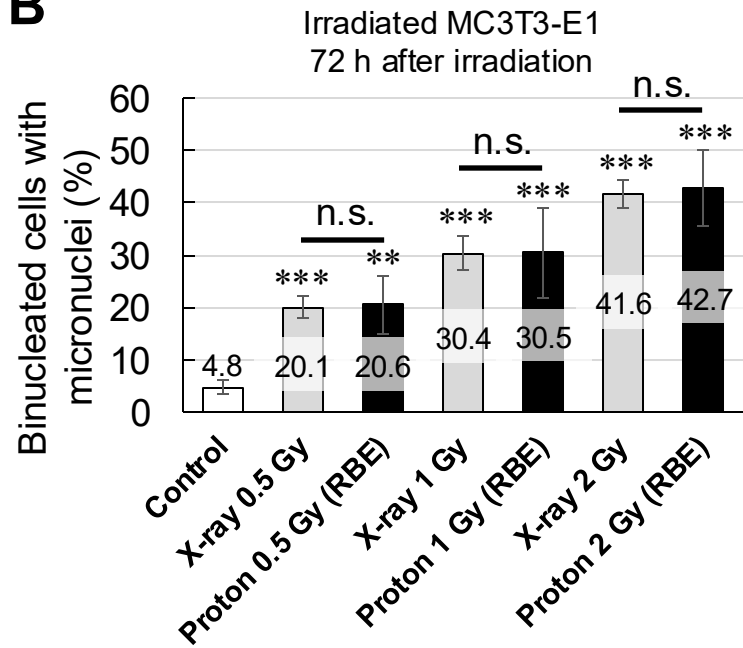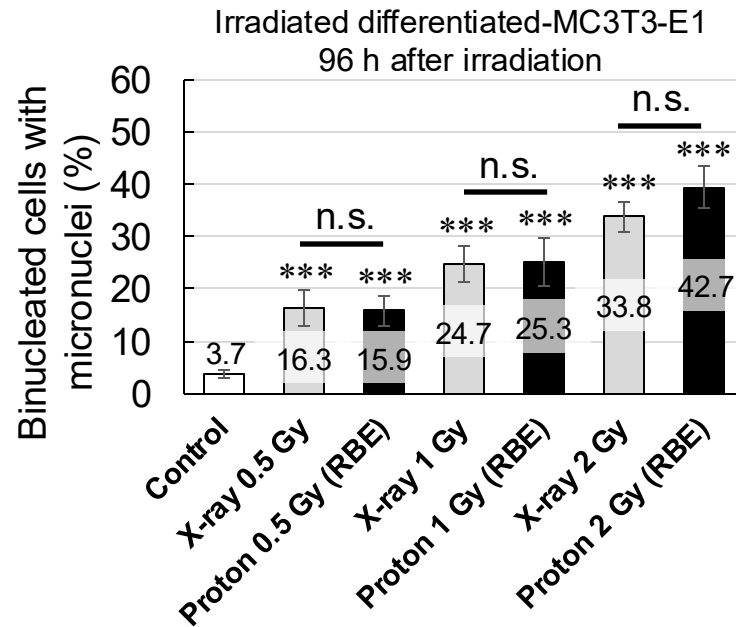

\*\* p<0.01, \*\*\* p<0.001, compared to the control

Supplement: Supplementary file 2 — Supplementary Data 2. Direct effects of irradiation on bone cells. (A) Percentage of 53BP1-positive nuclei at 1 h postirradiation in MC3T3-E1, differentiated MC3T3-E1, and RAW264.7 cells. (B) Percentage of micronucleated binucleate cells at 72 or 96 h postirradiation. RAW264.7 cells do not divide, so micronucleus assays have not been performed. *P < .05, **P < .01, ***P < .001 compared to nonirradiated controls [file mmc2.pdf]

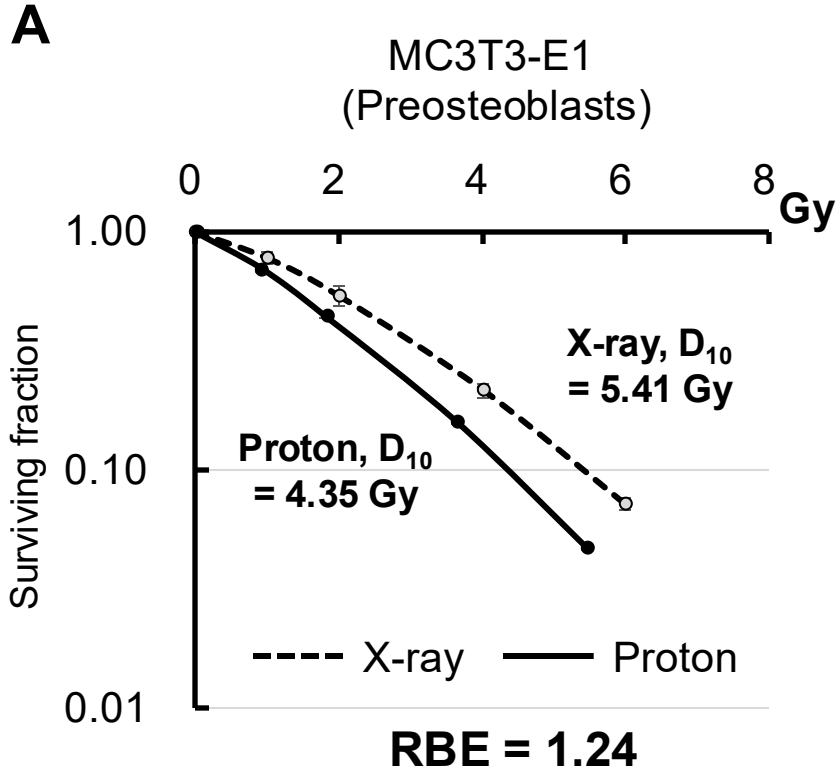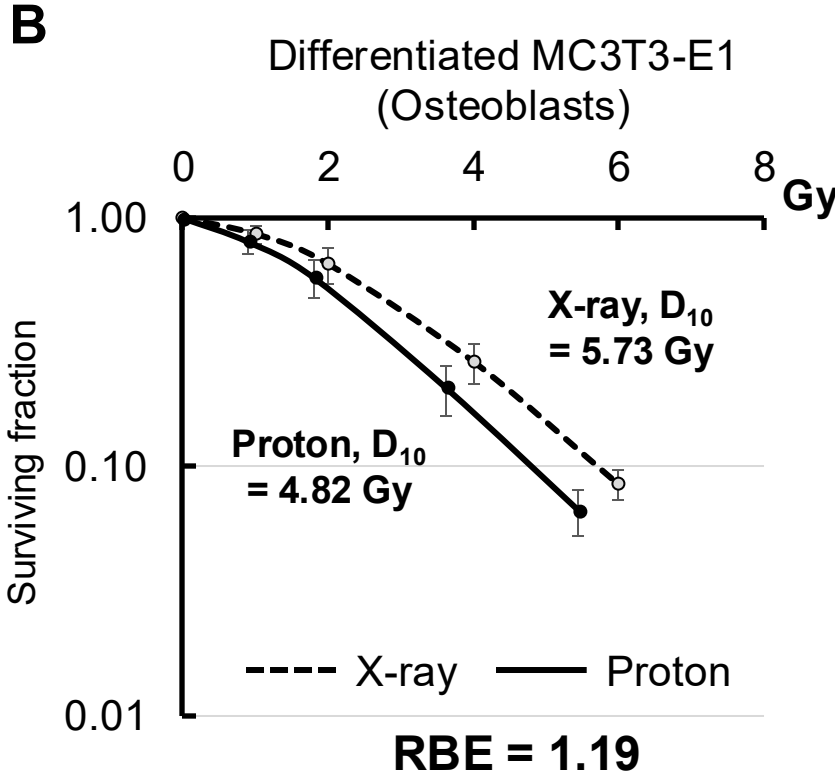

Supplement: Supplementary file 3 — Supplementary Data 3. Clonogenic survival of preosteoblasts and osteoblasts after irradiation. (A) MC3T3-E1 (preosteoblasts) and (B) differentiated MC3T3-E1 (osteoblasts) were exposed to x-ray or proton-irradiation at doses ranging from 0.5 to 6 Gy. Clonogenic survival was assessed by colony formation assay. Both cell types showed a dose-dependent decrease in surviving fraction following irradiation. The calculated D₁₀ values were 5.41 Gy (x-ray) and 4.35 Gy (proton) for preosteoblasts, and 5.73 Gy (x-ray) and 4.82 Gy (proton) for osteoblasts. The relative biological effectiveness (RBE) of protons compared to x-rays was estimated to be 1.24 for preosteoblasts and 1.19 for osteoblasts [file mmc3.pdf]
